# P-1745. Development and Use of a System Antimicrobial Stewardship Dashboard

**DOI:** 10.1093/ofid/ofae631.1908

**Published:** 2025-01-29

**Authors:** Jaime Borkowski, Erin Weslander, William Justin Moore, Nicole McGuire, Megha Tatti, Michael Postelnick

**Affiliations:** NM Delnor Hospital, Geneva, Illinois; Northwestern Memorial Hospital, Chicago, Illinois; Northwestern Medicine, Chicago, Illinois; Northwestern Medicine, Chicago, Illinois; Northwestern Medicine, Chicago, Illinois; Northwestern Medicine, Chicago, Illinois

## Abstract

**Background:**

Obtaining data has been an ongoing challenge for antimicrobial stewardship programs. With the creation and recent update of the Joint Commission antimicrobial stewardship standards, an increased focus has been placed on acting on relevant data to improve antimicrobial use. We describe the development and use of a systemwide dashboard to help track meaningful outcomes and identify areas of opportunity for our Antimicrobial and Diagnostic Stewardship Program (ADSP).

**Figure:**
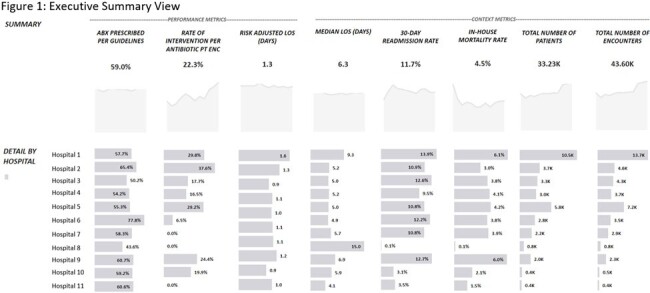

**Methods:**

A list of all potential pieces of data we may want to be able to compare was made, including demographic information, metrics, and important outcomes. Examples of dashboards already in use in our organization were reviewed to see what types of metrics were included and how they were displayed. The Enterprise Data Warehouse was used to pull data out of the electronic health records within Epic® and to house the dashboard.

**Figure:**
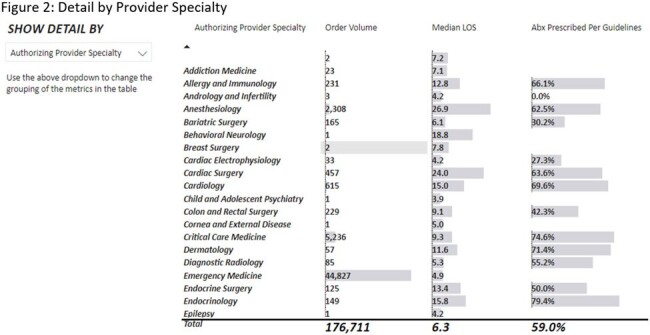

**Results:**

The dashboard contains three main views. The default view is the executive summary. This view contains performance and outcome metrics. These include adherence to guidelines, length of stay (LOS), 30-day readmission rate, and mortality rate. There is also a section below the overall summary that breaks out site-specific data. The second view includes population demographics and a breakdown of provider- and specialty-specific outcomes. The third view includes graphic representations of more granular detail broken out by treatment indication. In addition, we are able to filter each view by various details such as date, the ordering department, antimicrobial, treatment indication, and ADSP team intervention. One example of how we have used this dashboard so far is in the assessment of a systemwide initiative to improve empiric antibiotic prescribing for urinary tract infections. We were able to see increased guideline-consistent prescribing, decreased need for interventions, and no significant increase in LOS, readmission or mortality.

**Figure:**
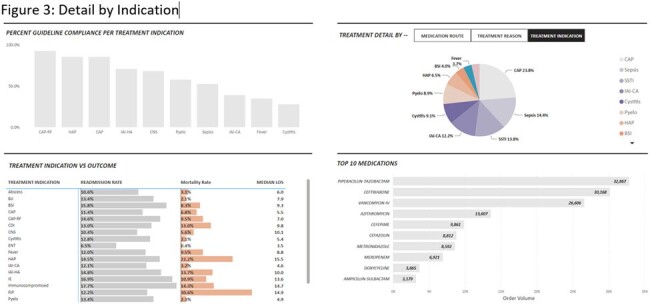

**Conclusion:**

This dashboard allows us to obtain data that would have previously required much time to gather and analyze. The inclusion of important patient-centered outcomes allows us to see the impact of ADSP interventions on our patients. It also aids in obtaining multiple pieces of data that TJC has outlined in the Antimicrobial Stewardship Standard.

**Figure:**
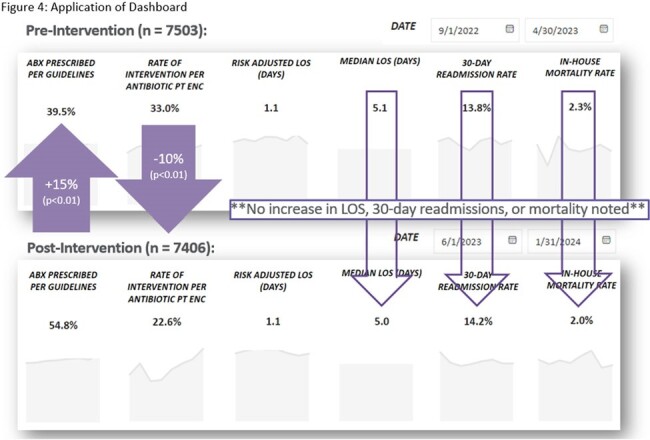

This graphic shows an example of how we are able to use our dashboard. Here we show a pre-post review of outcomes related to a systemwide initiative targeting empiric treatment of urinary tract infections. We saw an increase in guideline-consistent prescribing, a decreased need for ADSP intervention, and no significant increase in important outcomes including length of stay, readmission rate, or mortality (p>0.05 for readmission and mortality).

**Disclosures:**

**All Authors**: No reported disclosures

